# Implementing the Vision and Moving Forward

**Published:** 2009-12-15

**Authors:** Samuel F. Posner

*Preventing Chronic Disease* (*PCD*), the first issue of which was published in January 2004, is the product of Dr Lynne Wilcox's vision to create a journal that intersects research programs, practice, and policy. With the support of Dr James Marks, then the director of the National Center for Chronic Disease Prevention and Health Promotion, Dr Wilcox created a peer-reviewed publication dedicated to communicating multiple perspectives of public health professionals, from academics to advocacy groups to practitioners working in the field. The vision was, and is, for *PCD* to be published by but editorially independent of the Centers for Disease Control and Prevention (CDC).

Dr Wilcox anticipated a future in which technology could be used innovatively in publishing. *PCD* is CDC's first Web-only journal, a format that is becoming more common among scientific and academic publishers. *PCD* is also an open-access journal; its content is available free of charge and free of copyright and licensing restrictions. The only constraint on reproduction and distribution is *PCD*'s request that authors be properly acknowledged and cited.

The inclusion of multiple perspectives is central to *PCD*. The journal includes both peer-reviewed and non–peer-reviewed articles. The peer-reviewed articles fit the traditional academic and research models. Articles with nontraditional criteria, such as Tools and Techniques, focus on professional development. Regardless of article type, the journal requires authors to write in plain language and avoid technical terms and jargon to promote understanding to the broadest possible audience. These editorial policies continue to support *PCD*'s vision of inclusion and representation of traditional and nontraditional perspectives.

Dr Wilcox realized the value of the international community's contributions in understanding and addressing challenges in public health — to think globally for local action. Public health professionals from Canada and Mexico serve on *PCD*'s editorial board; these 2 nations represent the 2 largest segments of readers outside of the United States. Dr Wilcox implemented the translation of abstracts and full-text articles in Spanish, French, and traditional and simplified Chinese.

Although *PCD* accepts submissions on any chronic disease-related topic at any time during the year, the journal has also collaborated with guest editors on special themed collections. Each collection has covered different aspects of public health, including prevention, health promotion, interventions, disparities, and stakeholder engagement. These collections helped establish *PCD* as a venue for discourse on a range of public health topics.

By the end of its 5th year in 2008, the journal had a permanent staff of 13. *PCD* boasted approximately 15,000 subscribers, approximately 500 published articles, indexing in PubMed and PubMed Central, innovative technology, editorial decisions based on open and free communication of ideas, international recognition, and the exploration of new theories and concepts.

When Dr Wilcox retired from the United States Public Health Service in August 2008, 2 dedicated and capable scientists stepped up to serve *PCD* as acting editors in chief: Dr Marta Gwinn and Dr Susan Chu. Both had to hit the ground running, inheriting an existing production schedule, a backlog of manuscripts, a calendar of planned issues, and guest editors. Dr Gwinn and Dr Chu effectively navigated the editorial and leadership challenges. We thank them for their outstanding leadership to help *PCD* continue to grow and thrive.

## Moving Forward

In taking the helm of the journal as its permanent editor in chief, I want to ensure that *PCD* continues the same innovative spirit envisioned by Dr Wilcox and Dr Marks. *PCD* will not rest on its years of growth and accomplishments. Our tradition is to grow and meet challenges, and we are already planning several enhancements.

The journal has long hoped to publish 6 times per year, and we are now able to realize that goal. The rate of submissions to *PCD* is increasing, and our acceptance rate is decreasing. Both forces have improved quality. Our new schedule will allow us to manage the increased volume of accepted articles and offer faster turnaround from date of acceptance to date of publication.

The journal will continue to promote technologic innovations. We have added RSS feeds and multimedia content — including video files, audio files, animations, and video interviews. This year, we plan to add a "Post a Comment" feature, which will allow readers to offer feedback and stimulate discussion in a public forum. We expect to publish comments within 24 hours of posting. Facilitating dialogue is central to *PCD*'s mission.

Our international outreach will also continue. We now publish abstracts in Spanish, plus 2 full-text articles in Spanish related to health topics of interest to the Hispanic community, and we hope to translate more full-text articles into Spanish through partnership opportunities. *PCD* will continue its collaboration with counterparts at the Mexico Ministry of Health's General Directorate of Health Promotion, who published 2 articles in our January 2009 issue. We have also begun collaborating with the editors of *Chronic Diseases in Canada*, a peer-reviewed scientific journal published quarterly by the Public Health Agency of Canada. One topic of discussion has centered on copublishing opportunities.

We now want to know more about what our readers expect from us. We plan to survey *PCD*'s 15,000 subscribers to learn more about their interests, the topics in *PCD* that have been most helpful, and the kinds of features *PCD* should include. We also plan to examine different measures of impact, including subscriptions, submissions, downloads, highly accessed articles, and citations of *PCD* articles.

Finally, we plan to make a few changes to our content and to our strategy of collaborating with guest editors. A new type of article is now available to authors. The Brief is a short (no more than 1,000 words), peer-reviewed research report that will benefit authors who wish to submit their report in a condensed form for quick review and publication. Criteria for the Brief are on our Web site (http://www.cdc.gov/pcd/for_authors/types_of_articles.htm#Brief).

Our strategy of collaborating with guest editors on special themed collections not only helped the journal to gain more exposure with internal and external organizations and partners but also demonstrated that the journal is a venue for publishing on varied content areas. The strategy was critical to establishing the journal's presence in the field. Our priority now is to accommodate the increasing number of submissions, so we will no longer regularly collaborate with a guest editor on themed collections.

The progress and success of *PCD* would not have happened without its experienced and dedicated staff, the authors, reviewers, and readers. The invaluable work of our peer reviewers helps set the highest quality standards for *PCD*. The editorial board has provided thoughtful guidance and support since *PCD*'s inception. We will continue to work together to ensure that *PCD* continues to publish timely, high-quality, and relevant reports for the public health professionals we serve.

In implementing these changes, I believe that we are rededicating ourselves to the mission of the journal as envisioned by its founding editor. *PCD* will continue to promote dialogue between public health researchers and practitioners, encourage interdisciplinary research, and offer a venue for the many voices and perspectives in public health. I look forward to receiving feedback from our readers as we implement these enhancements.

